# Effects of high-fat diet and *Apoe* deficiency on retinal structure and function in mice

**DOI:** 10.1038/s41598-020-75576-7

**Published:** 2020-11-02

**Authors:** Xiupeng Cao, Yatu Guo, Yuchuan Wang, Hao Wang, Dong Liu, Yibo Gong, Jue Wang, Xia Chen, Wei Zhang

**Affiliations:** 1 Tianjin Medical University, Tianjin, China; 2 Tianjin Key Lab of Ophthalmology and Visual Science, Tianjin Eye Hospital, Tianjin Eye Institute, Tianjin, China; 3 Nankai University Affiliated Eye Hospital, Tianjin, China; 4 State Key Laboratory of Food Nutrition and Safety, Tianjin University of Science and Technology, Tianjin, China

**Keywords:** Diseases, Health care, Medical research

## Abstract

To investigate the effects of a high-fat diet (HFD) and apolipoprotein E (*Apoe*) deficiency on retinal structure and function in mice. *Apoe* KO mice and wild-type C57BL/6J mice were given a low-fat diet (LFD) or a HFD for 32 weeks. Blood glucose, serum lipids, body weight and visceral fat weight were evaluated. Retinal sterol quantification was carried out by isotope dilution gas chromatography-mass spectrometry. The cholesterol metabolism related genes SCAP-SREBP expressions were detected by qRT-PCR. Retinal function was recorded using an electroretinogram. The thickness of each layer of the retina was measured by optical coherence tomography. Fundus fluorescein angiography was performed to detect retinal vasculature changes. Immunohistochemical staining was used to determine the expression of NF-κB, TNF-α and VEGFR2 in the retina among HFD, HFD *Apoe*^−/−^, LFD *Apoe*^−/−^ and WT mice retinas. HFD feeding caused the mice to gain weight and develop hypercholesterinemia, while *Apoe*^−/−^ abnormalities also affected blood lipid metabolism. Both HFD and *Apoe* deficiency elevated retinal cholesterol, especially in the HFD *Apoe*^−/−^ mice. No up-regulated expression of SCAP-SREBP was observed as a negative regulator. Impaired retinal functions, thinning retinas and abnormal retinal vasculature were observed in the peripheral retinas of the HFD and *Apoe*^−/−^ mice compared with those in the normal chow group, particularly in the HFD *Apoe*^−/−^ mice. Moreover, the expression of NF-κB in the retinas of the HFD and *Apoe*^−/−^ mice was increased, together with upregulated TNF-α mRNA levels and TNF-α expression in the layer of retinal ganglion cells of the peripheral retina. At the same time, the expression level of VEGFR2 was elevated in the intervention groups, most notably in HFD *Apoe*^−/−^ mice. HFD or *Apoe* gene deletion had certain adverse effects on retinal function and structure, which were far below the combined factors and induced harm to the retina. Furthermore, HFD caused retinal ischemia and hypoxia. Additionally, *Apoe* abnormality increased susceptibility to ischemia. These changes upregulated NF-κB expression in ganglion cells and activated downstream TNF-α. Simultaneously, they activated VEGFR2, accelerating angiogenesis and vascular permeability. All of the aforementioned outcomes initiated inflammatory responses to trigger ganglion cell apoptosis and aggravate retinal neovascularization.

## Introduction

With changes in modern lifestyles, the number of people with overnutrition and little exercise is gradually increasing, leading to significant increases in obesity and hypercholesterolemia^1^. It is generally accepted that metabolic syndrome (MetS)^2^ is involved in the pathogenesis of a variety of diseases, including vascular diseases and neurodegenerative diseases^3^. As a part of visual neural system, the retina plays an important physiological function in visual performance. Another structural feature of retina is that it is rich in blood vessels. The central retinal artery passing through the optic disc is divided into four branches, which travel inside the retinal nerve fiber layer and gradually distribute to the peripheral area. The retina is a layered structure with several interconnected neurons, such as the outer nuclear layer, containing the nuclei of the photoreceptor cells, and it connects to the ganglion cell layer (GCL) through the bipolar cells, which are located in the inner nuclear layer (INL). The nerve fibers (axons) from the ganglion cells converge toward the optic disc and enter the brain through the optic nerve. Therefore, structural abnormalities of the retina affect signal transduction and thus injure visual function^4^. When peripheral retinopathy occurs, including abnormal blood supply and ganglion cell apoptosis, the visual signals of rod cells are limited to transmitting to the optic nerves, causing night blindness^5^. Furthermore, the risk of peripheral retinal detachment increases due to peripheral retinopathy.

Apolipoprotein E (APOE) is necessary to help remove cholesterol-rich lipoprotein from the circulation. In addition to hyperlipidemia caused by HFD in C57BL/6J mice, normal-diet *Apoe*^−/−^ mice can also have spontaneous hypercholesterolemia^6^ because *Apoe*^−/−^ mice have the genetic background of a cholesterol metabolism disorder^7^. Compared to normal mice, central nervous system (CNS) neurons in *Apoe*^−/−^ mice are more sensitive to ischemic injury^8^. Therefore, the *Apoe* gene has a certain protective effect on the nerves and blood vessels and even the retinal ganglion cells (RGCs)^9^. The effects of HFD and *Apoe* gene deletion on the retina are both popular research topics, but it is rare to investigate their combined effects on the retina. We used HFD *Apoe*^−/−^ mice to investigate the effects of double threats on the structure and function of the retina, as well as the underlying mechanisms.

## Results

### Chronic HFD and/or *Apoe* deficiency leads to serum and retinal lipid metabolic abnormality of mice

**Figure 1:**
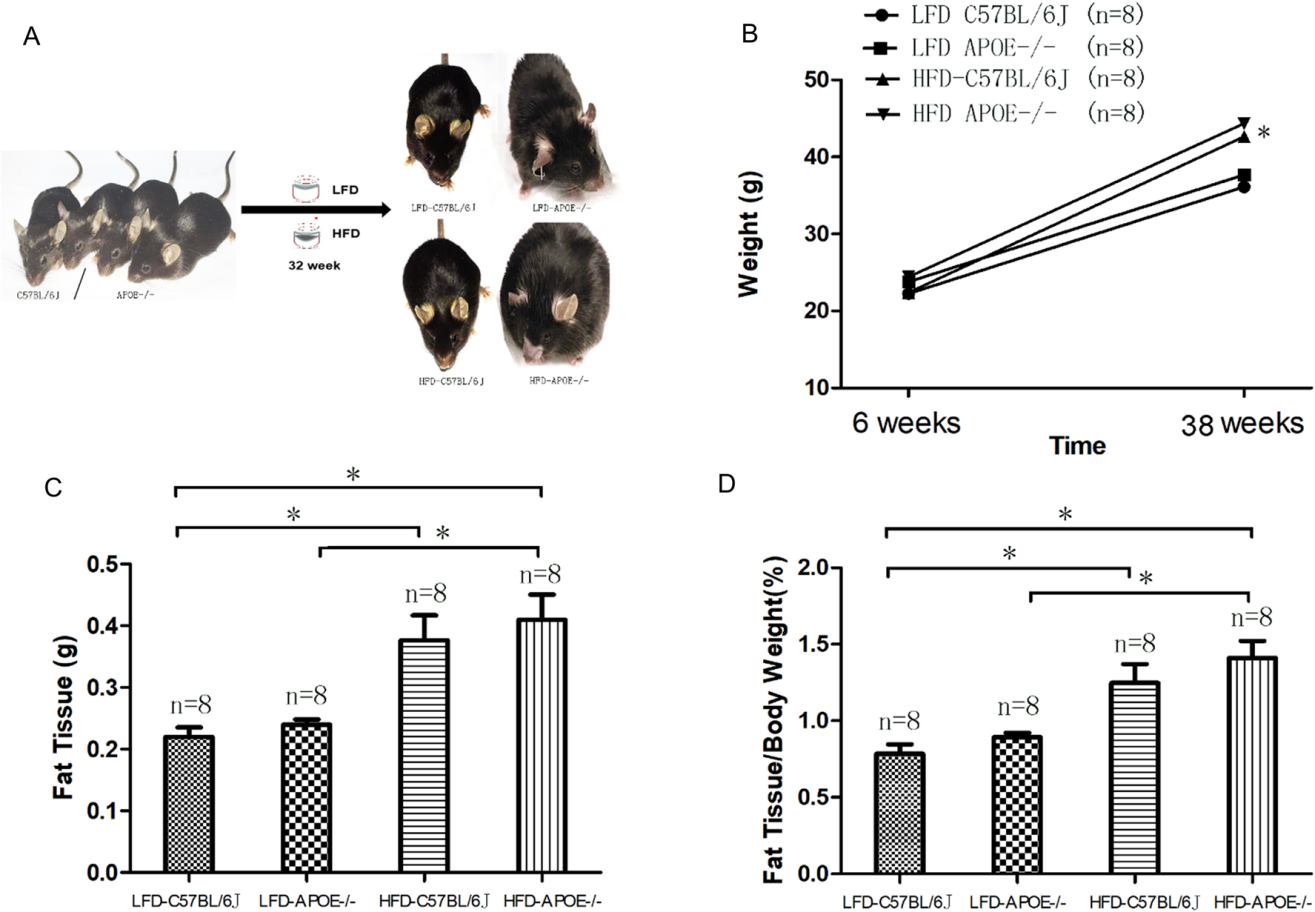
Glucose/lipid metabolic abnormality in wild-type and *Apoe−/−* mice fed with HFD or LFD. **A** Wild-type and *Apoe*−/− mice were divided in two groups: high-fat diet (HFD, 18.4% fat, 17% proteins, 66.6% carbohydrates) and low-fat diet (LFD, control, 3.4% fat, 17% proteins, 81.6% carbohydrates. Pictures taken from Dr Guo’s Lab), **B**,**C** Changes in the body weight and amount of visceral adipose tissue (VAT) in four groups, **D** Changes in body fat percentage standard(Fat issue/body weight) in four groups. Error bars were the mean ± SEM. *P < 0.05.

Starting from the 6th week, HFD and LFD were given to *Apoe*^−/−^ mice (HFD *Apoe*^−/−^ and LFD *Apoe*^−/−^) and C57BL/6J mice (HFD C57BL/6J and LFD C57BL/6J), in which the LFD C57BL/6J group was the control group. After the 32nd week, the body weight and amount of visceral adipose tissue (VAT) of the mice in the HFD groups were higher than those in the LFD groups (LFD C57BL/6J: 36.32 ± 0.46 g, n = 8; HFD C57BL/6J: 44.36 ± 2.06 g, n = 8; HFD *Apoe*^−/−^: 45.69 ± 3.42 g, n = 8; *P*_2_ < 0.0001; *P*_3_ < 0.0001, as shown in Fig. 1B,C). Assessment of mouse obesity potency was based on body fat percentage standard (fat tissue/body weight). Obesity was shown in mice with HFD (Fig. 1D). Serum lipid analysis showed that serum total cholesterol (TC) in the LFD *Apoe*^−/−^, HFD C57BL/6J and HFD *Apoe*^−/−^ mice was increased (LFD C57BL/6J: 4.33 ± 0.69 mmol/L, n = 8; LFD *Apoe*^−/−^: 11.60 ± 2.04 mmol/L, n = 8; HFD C57BL/6J: 10.55 ± 0.77 mmol/L, n = 8; HFD *Apoe*^−/−^: 14.24 ± 1.25 mmol/L, n = 8), and compared with the control group (LFD C57BL/6J), the differences were statistically significant (***P***_**1**_: LFD C57BL/6J vs LFD *Apoe*^−/−^; ***P***_**2**_: LFD C57BL/6J vs HFD C57BL/6J; ***P***_**3**_: LFD C57BL/6J vs HFD *Apoe*^−/−^ *P*_1_, *P*_2_*, P*_3_ < 0.0001). The levels of triglycerides and non-high-density lipoprotein cholesterol (non-HDL-C) were also increased, but the level of HDL-C did not change significantly (as shown in Table 1), while the average fasting blood glucose levels were significantly increased in the HFD and *Apoe*^−/−^mice compared with the normal control group (LFD C57BL/6J: 5.59 ± 0.72 mmol/L, n = 8; LFD *Apoe*^−/−^: 6.30 ± 0.32 mmol/L, n = 8; HFD C57BL/6J: 6.50 ± 0.3 mmol/L, n = 8; HFD *Apoe*^−/−^: 7.85 ± 1.91 mmol/L, n = 8). The LFD *Apoe*^−/−^, HFD C57BL/6J and HFD *Apoe*^−/−^ mice all had impared fasting glucose due to high average fasting blood glucose (> 6.11 mmol/L). These results indicated that *Apoe*^−/−^ and HFD both led to abnormalities in TC and blood glucose in the mice. HFD also caused increases in body weight and VAT in the mice (including wild-type and *Apoe*^−/−^ mice).

**Table 1:** Analysis of serum lipids in mice of each group.

n = 8	A.LFD-C57BL/6J	B.LFD-APOE−/−	C.HFD-C57BL/6J	D.HFD-APOE−/−	*P* value		
					A versus B	A versus C	A versus D
TC	4.33 ± 0.69	11.6 ± 2.04	10.55 ± 0.77	14.24 ± 1.25	< 0.0001	< 0.0001	< 0.0001
TG	5.31 ± 0.73	6.18 ± 0.60	6.65 ± 0.37	6.96 ± 0.54	0.037	0.001	< 0.0001
HDL-C	1.87 ± 0.34	1.75 ± 0.34	1.61 ± 0.24	1.58 ± 0.24	0.859	0.506	0.378
Non-HDL-C	2.96 ± 0.35	9.89 ± 0.94	11.15 ± 1.15	13.26 ± 0.80	< 0.0001	< 0.0001	< 0.0001

**Figure 2:**
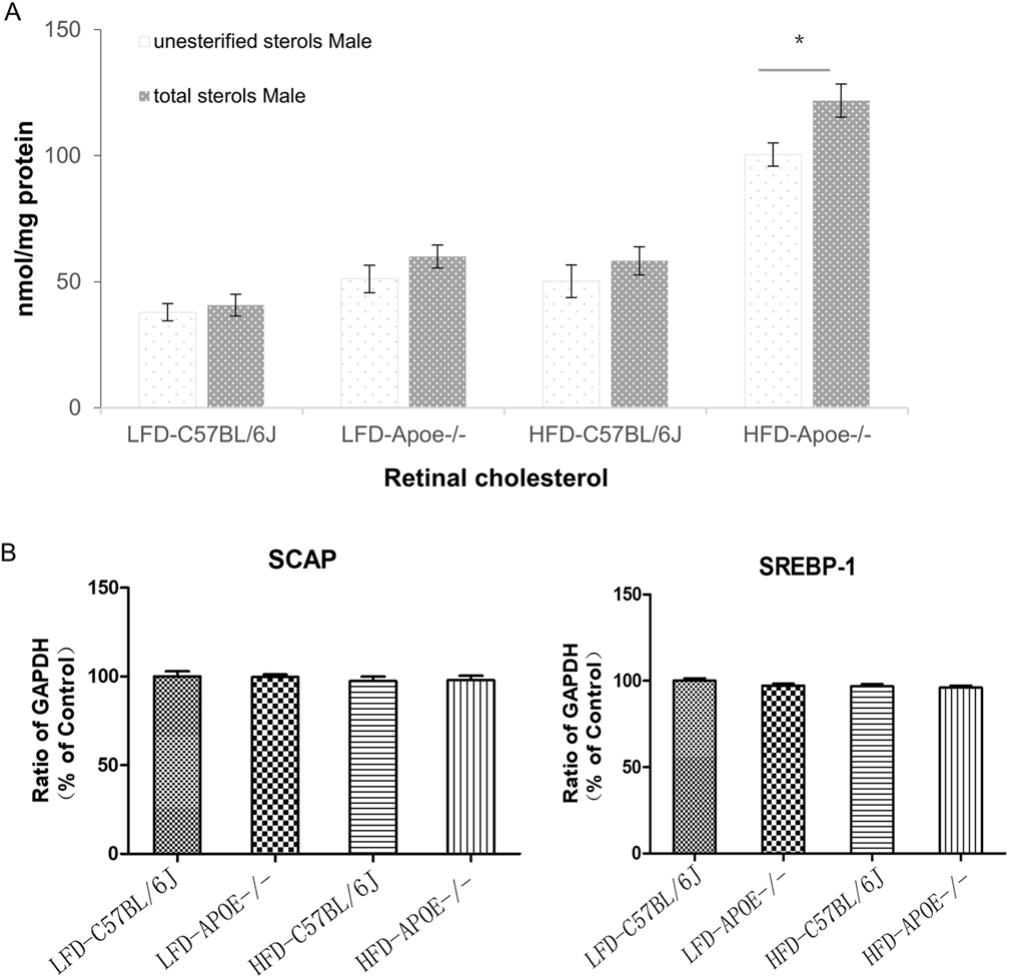
The sterols and the expressions of lipid metabolism related genes in mice retina. **A** Retinal sterol quantifications. Results are mean ± SD of the measurements in individual mice (4 animals/genotype/sex; number of mice equals the number of retinas); animals were 38-week-old. Statistical significance was assessed by two-way ANOVA with Bonferroni correction. Asterisk indicate statistically significant differences between sexes of the same genotype. nError bars were the mean ± SEM. *P < 0.05. **B** The expressions of lipid metabolism related genes in mice retina. Sterol cleavage activating protein (SCAP), sterol regulatory element binding protein (SREBP-1) in *Apoe*−/− mice and C57BL6/J with HFD and LFD mice. GAPDH was used as the internal reference of PCR. The mean value and standard deviation of 8 cDNA samples were shown in each histogram, and LFD-C57BL/6J was used as the control.

Because the levels of esterified cholesterol were very low in mice retinas, unesterified (free) and total cholesterol were evaluated. The levels of retinal cholesterol (unesterified and total cholesterol) were increased compared to wild-type animals although they did not achieve statistical significance. In HFD *Apoe−/−* mice, the retinal cholesterol contents were much higher than other three groups. [LFD C57BL/6J (nmol/mg): unesterified cholesterol 37.89 ± 3.38; Total cholesterol 40.76 ± 4.32; LFD *Apoe−/−* (nmol/mg): unesterified cholesterol 51.10 ± 5.47; Total cholesterol 60.03 ± 4.53 HFD C57BL/6J (nmol/mg): unesterified cholesterol 50.20 ± 6.45; Total cholesterol 58.32 ± 5.56; HFD *Apoe−/−* (nmol/mg): unesterified cholesterol 100.43 ± 4.65; Total cholesterol 121.85 ± 6.56, Two-way ANOVA F[3.64] = 50.66 *P* = 0.000 < 0.05 as shown in Fig. 2A).

To investigate the gene regulation of lipid metabolism in the retina, we performed RT-PCR analysis on sterol cleavage activating protein (SCAP) and sterol regulatory element binding protein (SREBP) in the retina of each group of mice. SCAP is an endoplasmic reticulum membrane binding protein and an intracellular sterol receptor. SCAP forms a complex with SREBP after endoplasmic reticulum synthesis. The results revealed that there had no effect on the upregulation of these lipid metabolism related genes (SCAP and SREBP-1) when comparing the high-fat diet and *Apoe* knockout mice to the normal control mice (Fig. 2B, One-way ANOVA *P* > 0.05).

### Chronic HFD decreased the thickness of the peripheral retina, and chronic HFD and *Apoe* deficiency make the peripheral retina even thinner

**Figure 3:**
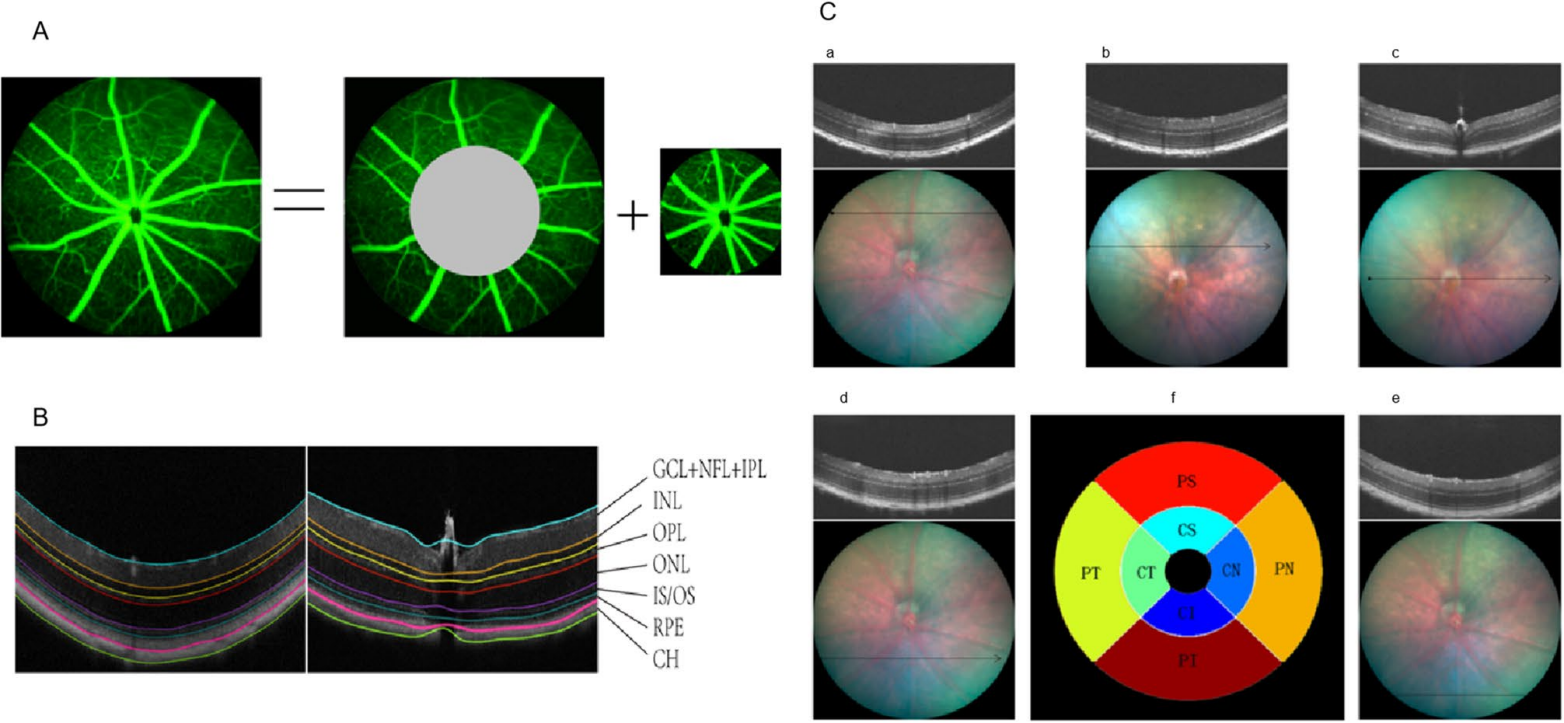
Retinal fundus fluorescein angiography and fundus photography corresponding with the cross-sectional OCT images. **A** FFA images include central and peripheral region. The central region is defined as three times the diameter of the optic disc, the rest of the retina considered as the peripheral region. **B** Seven targeted retinal layer boundaries in SD-OCT scan. **C** The upper (a), upper-middle (b), middle (c), middle-lower (d), and lower areas (e) of the retina were scanned. The scanning lines positioned 3 PD and 1.5PD from the optic disc separately. When measuring the central area, a full-length retinal scan across the middle of disc (e); The reproducibility measures were computed in the eight regions shown above, namely, the CS, CN, CI, CT, PS, PN, PI, and PT regions. The inner, middle, and outer circles are 0.2, 0.6, and 1.2 mm in diameter, respectively (f).

**Figure 4:**
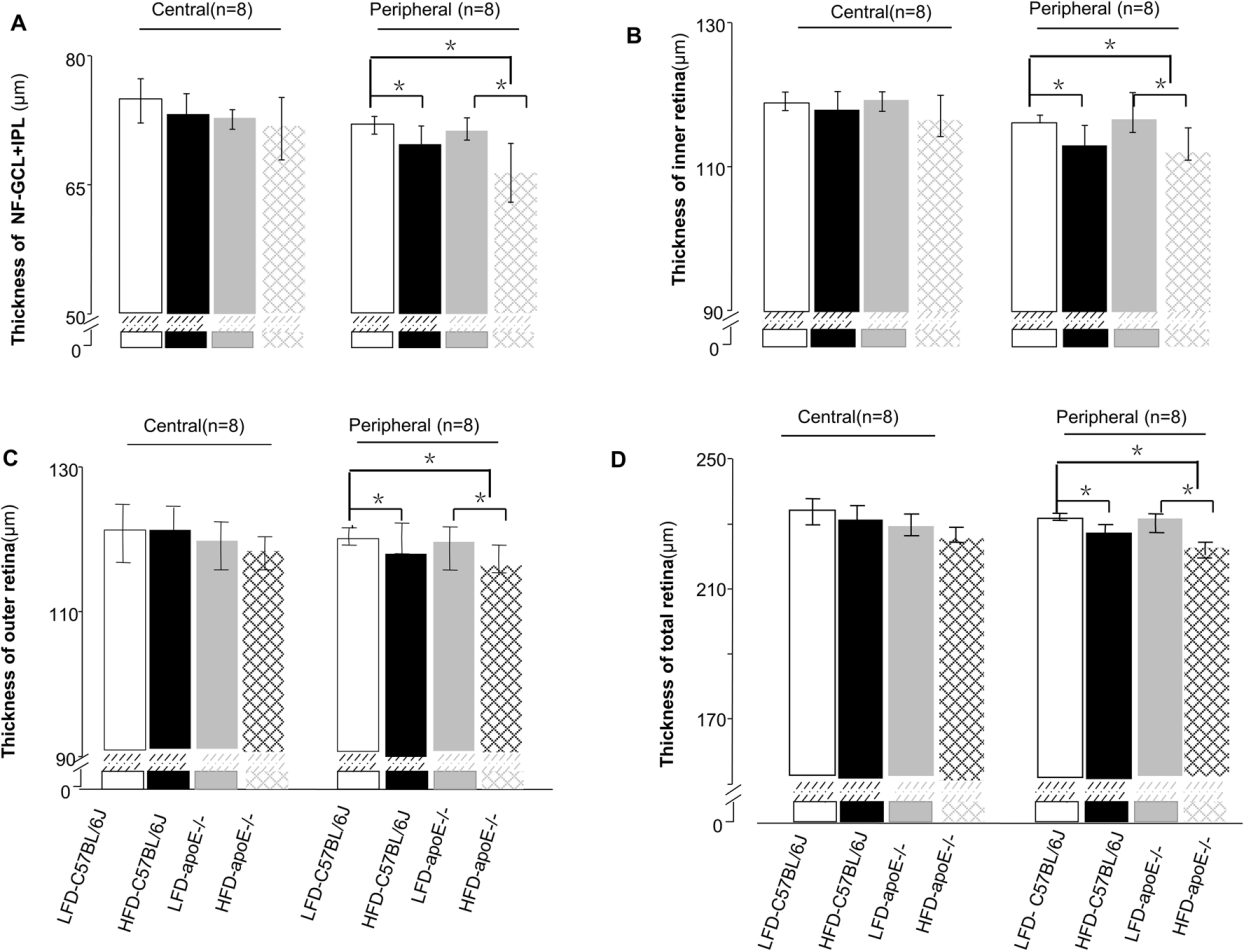
Assessments for thickness of different retinal layers. **A**–**D** Thickness of the NFL + GCL + GCL, inner retina, outer retina, and total retina(the peripheral area and central area). Data were shown as mean ± SEM. (n = 8). *P < 0.05.

SD-OCT was performed to evaluate the thickness of the mouse retina. Due to difficulty in distinguishing the three layers—NFL, GCL and IPL—during OCT assessment, they are often considered a composite layer (NFL − GCL + IPL). The retinal NFL + GCL + IPL, inner retina, outer retina and total retinal thickness (TRT) were evaluated (Fig. 3B). We found that there were no effects of the HFD and LFD on the thickness of each layer in the central retina for the wild-type mice and *Apoe*^−/−^ mice (Fig. 4A–D).

However, for the peripheral area, the thickness of the NFL + GCL + IPL of the HFD C57BL/6J mice and HFD *Apoe*^−/−^ mice was significantly thinner than that of the LFD C57BL/6J mice, as shown in Fig. 4A (HFD C57BL/6J: 69.53 ± 1.54, n = 8; HFD *Apoe*^−/−^: 66.43 ± 1.52, n = 8; LFD C57BL/6J 71.38 ± 1.16, n = 8, one-way ANOVA: *P*_HFD-C57BL/6 vs control_ = 0.011, *P*_HFD *Apoe*−/− vs control_ < 0.001). The combined structure of the HFD *Apoe*^−/−^ mice was thinner than that of the LFD *Apoe*^−/−^ mice (66.43 ± 1.52, n = 8; 71.39 ± 1.16, n = 8, *P* < 0.001) and even thinner than in the HFD mice (*P* = 0.001). Although there was a trend toward a composite layer decrease, no significant change occurred in the thickness of the NFL + GCL + IPL layer of the LFD *Apoe*^−/−^ mice compared to the control group (71.39 ± 1.16, n = 8;71.96 ± 1.42, n = 8, *P* = 0.420). Additionally, thinning changes were observed in the HFD C57BL/6J mice and HFD *Apoe*^−/−^ mice in the other three layers (inner retina, outer retina and TRT) in the peripheral area (HFD C57BL/6J mice inner retina: 113.24 ± 1.31, n = 8; outer retina: 114.34 ± 0.97, n = 8; TRT: 228.56 ± 1.35, n = 8; HFD *Apoe*^−/−^ mice: inner retina: 112.92 ± 1.12, n = 8; outer retina: 114.34 ± 1.49, n = 8; TRT: 227.27 ± 1.41, n = 8). Compared with the control group, there were significant reductions in thickness in the HFD C57BL/6J mice and HFD *Apoe*^−/−^ mice (control group: inner retina: 115.23 ± 1.43, n = 8; outer retina: 116.51 ± 1.20, n = 8; TRT: 230.71 ± 1.70, n = 8; one-way ANOVA, *P* inner layer _HFD-C57BL/6 vs control_ = 0.024; *P*_HFD *Apoe*−/− vs control_ = 0.007; *P* outer layer _HFD-C57BL/6 vs control_ = 0.034; *P*_HFD *Apoe*−/− vs control_ = 0.007; *P* TRT_HFD-C57BL/6 vs control_ = 0.03; *P*_HFD *Apoe*−/− vs control_ < 0.001), but there were no differences in the thickness of the other three layers between the LFD *Apoe*^−/−^ mice and the control group (one-way ANOVA *P* > 0.05) (Fig. 4A–D).

**Figure 5:**
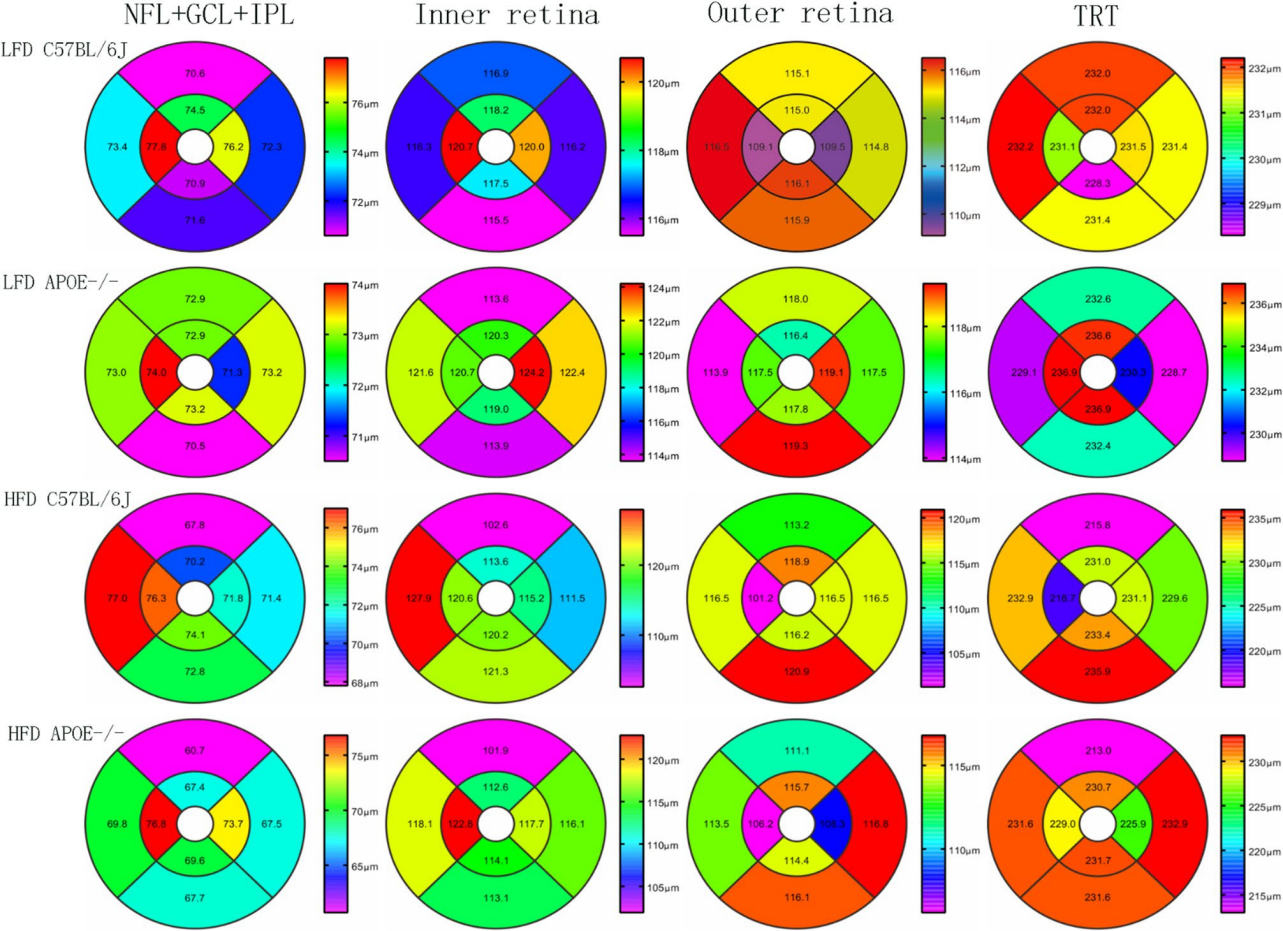
The representative heat map of retinal thickness. The thickness differences computed (mean [SD] μm) within the NFL + GCL + IPL (first line), the inner retina (second line), the outer retina (third line), and the total retinal thickness (last line) for LFD-C57BL/6J mice, LFD *Apoe−/−* mice, HFD-C57BL/6J mice and HFD *Apoe−/−* mice.

To explore more details, the mean thickness was then computed for each of the layers in eight regions, namely, the central superior (CS), central nasal (CN), central inferior (CI), central temporal (CT), paracentral superior (PS), paracentral nasal (PN), paracentral inferior (PI) and paracentral temporal (PT) regions (Fig. 3C(f)). The circles used were 0.2 mm, 0.6 mm and 1.2 mm in diameter, respectively. The value in each region of the heat map represents the mean thickness of the retinal area (Fig. 5).

These data indicated that the HFD alone could significantly reduce each layer’s thickness of the peripheral retina of the mice, especially for the composite layer (NFL + GCL + IPL). The hypercholesterolemia caused by *Apoe* gene knockout was not sufficient to change the thickness of the peripheral retina of the mice. However, double factors further reduced the thickness of the NFL + GCL + IPL in the peripheral retina.

### Chronic HFD and/or *Apoe* deficiency increased the capillary density and nonperfusion zone of the peripheral retina

**Figure 6:**
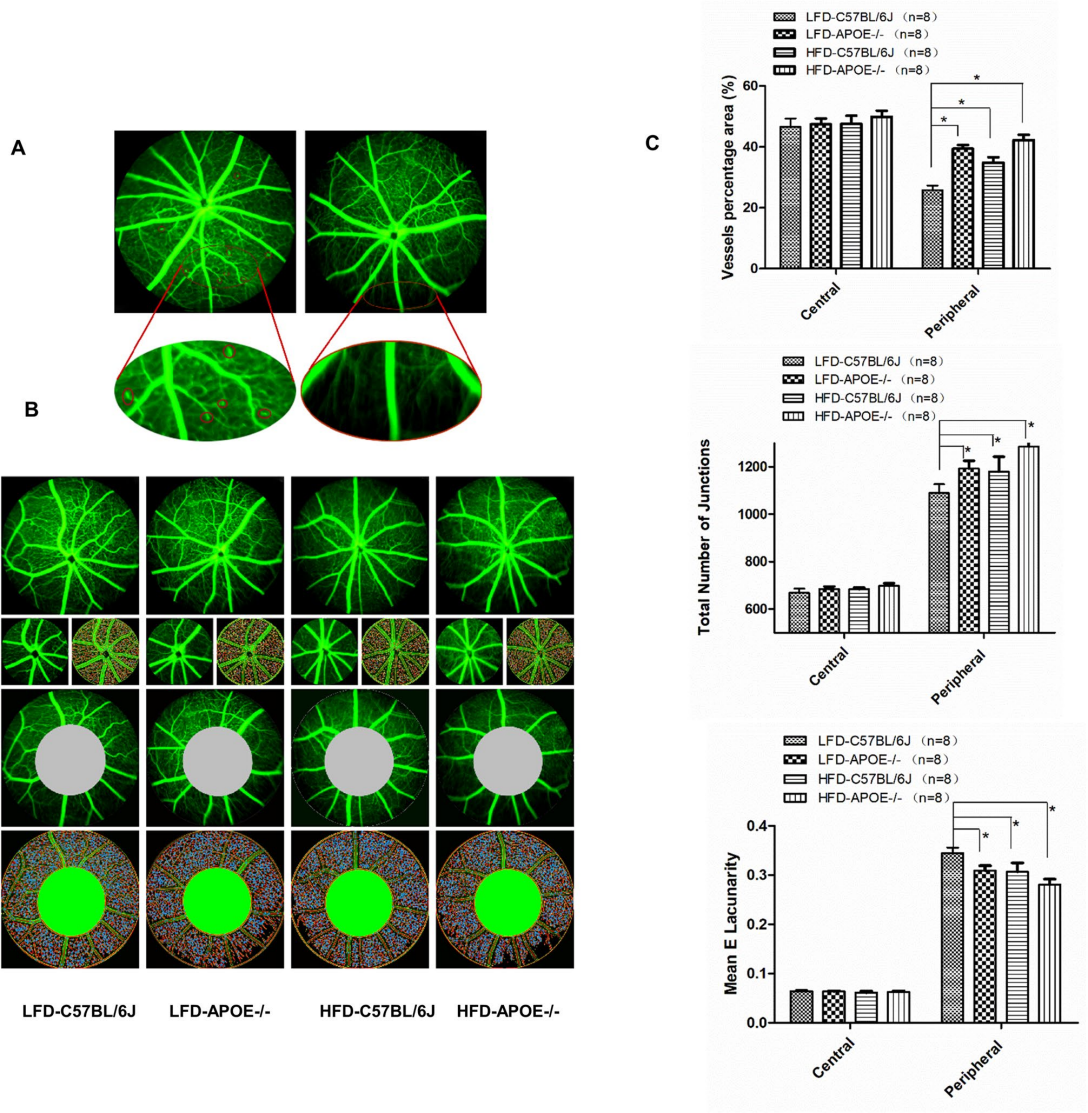
Quantification of the capillary density. **A** HFD *Apoe−/−* mice exhibit microaneurysm and capillary non-perfusion zone in peripheral retina. **B** Microvessel density was assessed in the central and peripheral retina. **C** No statistically significant differences among four groups was observed comparing the central concerning vessels density, number of junctions and Mean E Lacunaroty. However, comparing the same values in the peripheral retina we observed significant differences among four groups. Data were shown as mean ± SEM. (n = 8). *P < 0.05.

Fundus fluorescein angiography depicted subtle differences in retinal vasculature, such as retinal microaneurysms and capillary nonperfusion zones, in the HFD *Apoe*^−/−^ and HFD-C57BL/6J mice (Fig. 6A). Comparison of the vascularization of the peripheral and central retina revealed differences in vessel coverage between different areas in each group (Fig. 6B); therefore, we analyzed the vascular density in the peripheral retina and the central retina using Angio Tool software(National Institutes of Health National Cancer Institute, Gaithersburg, MD)^10^. Two regions of the retinal parenchyma were selected for microvessel density analysis (Fig. 6B). This analysis demonstrated that there was no significant difference in vessel density or number of junctions when analyzing the central retina among these four groups. Conversely, analysis of the peripheral retina showed significant differences in all three features, with all of them being significantly higher in the HFD *Apoe*^−/−^ mice, HFD C57BL/6J mice and LFD *Apoe*^−/−^ mice than in the control group (Fig. 6C) (HFD *Apoe*^−/−^ mice: vessel percentage (%): 42.22 ± 1.75, n = 8; branch points: 1285.38 ± 91.35, n = 8; HFD C57BL/6J mice: vessel percentage (%): 34.81 ± 1.74, n = 8; branch points: 1180.25 ± 62.49, n = 8; LFD *Apoe*^−/−^ mice: vessel percentage (%): 39.39 ± 1.22, n = 8; branch points: 1192.75 ± 33.37, n = 8; LFD C57BL/6J mice: vessel percentage (%): 25.81 ± 1.53, n = 8; branch points: 1089.63 ± 37.46, n = 8; one-way ANOVA *P* < 0.05 for all). These results indicate that hypercholesterolemia caused by HFD or *Apoe* gene deletion was sufficient to cause blood vessel abnormalities in the peripheral retina. Capillary density and nonperfusion zones were more significantly increased in the HFD *Apoe*^−/−^ mice than in the age-matched HFD C57BL/6J mice or LFD *Apoe*^−/−^ mice.

### Chronic HFD and/or *Apoe* deficiency impaired retinal function

**Figure 7:**
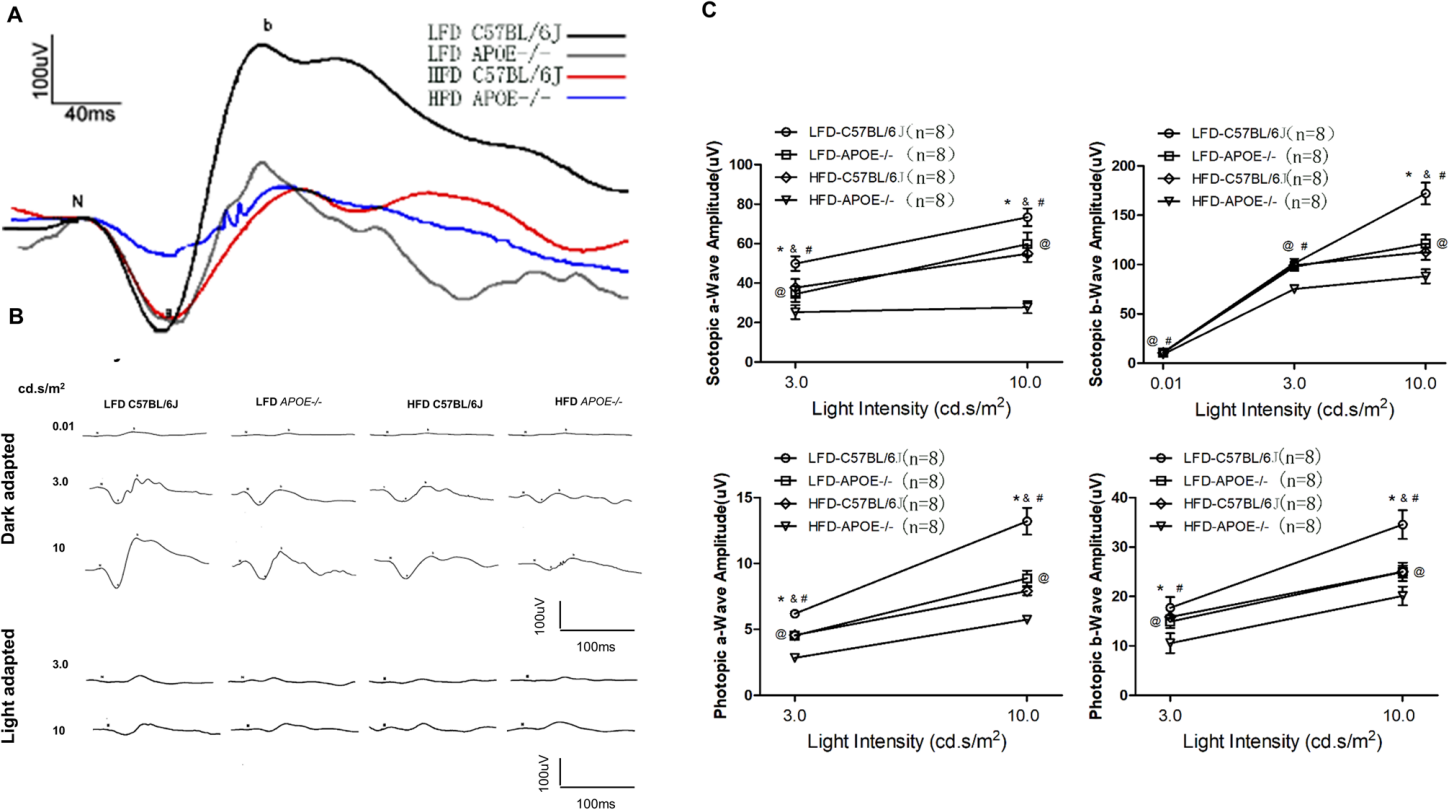
The effects of HFD and apoE deletion on retinal function. **A** Representative ERG plots for four groups in response to a light flash of 10 cd s/m^2^. **B** Representative ERG responses in the control, the LFD-*ApoE−/−*, the HFD and the HFD*-ApoE−/−* groups. **C** Intensity response curves of the 6 highest light stimulation intensities (0.1, 3, 10 cd s/m^2^) are presented. Results shown the mean ± SEM of the amplitudes (mV) and implicit times (ms) of a- and b-waves as a function of stimulus intensity. (n = 8; *P,0.05).

Chronic HFD and *Apoe* deletion were capable of inducing subtle changes in retinal structure, but it remains unknown whether these two factors could cause malfunction of the retina. Therefore, the retinal function in the mice was tested with ERG, and the impairment of retinal function was observed. Dark- and light-adapted ERGs were performed in the 32nd week. The amplitudes of the a-wave and b-wave, which represent the functions of the photoreceptors and bipolar cells, respectively (Table 2), were significantly decreased in the HFD *Apoe*^−/−^ mice, HFD-C57BL/6J mice and LFD *Apoe*^−/−^ mice—especially the HFD *Apoe*^−/−^ mice (Fig. 7A, B). Further, chronic HFD and *Apoe* deletion induced even more decreased amplitudes of the ERG a- and b-wave (Fig. 7C).

**Table 2:** Amplitude changes of a-wave and b-wave under different stimulus intensity under dark adaptation and light adaptation.

(μV) n = 8	A.LFD-C57BL/6J	B.LFD-APOE−/−	C.HFD-C57BL/6J	D.LFD-APOE −/−	*p* value		
					A versus B	A versus C	A versus D
**Dark adapt (cd s/m**^2^**)**							
0.01 b-wave	16.41 ± 1.65	11.11 ± 0.86	10.15 ± 1.05	8.69 ± 1.45	< 0.05	< 0.05	< 0.05
3.0 a-wave	50.43 ± 3.24	34.41 ± 3.89	36.3 ± 3.27	25.21 ± 2.90	< 0.05	< 0.05	< 0.05
3.0 b-wave	124.58 ± 9.92	88 ± 6.51	86.26 ± 5.82	68.41 ± 6.34	< 0.05	< 0.05	< 0.05
10.0 a-wave	110.35 ± 17.07	86.99 ± 7.93	83.11 ± 5.75	64.24 ± 7.02	< 0.05	< 0.05	< 0.05
10.0 b-wave	172.21 ± 10.3	121.21 ± 7.83	112.75 ± 7.04	83.11 ± 8.59	< 0.05	< 0.05	< 0.05
**Light adapt (cd s/m**^2^**)**							
3.0 a-wave	12.24 ± 0.96	8.9 ± 0.81	82.29 ± 0.77	4.54 ± 0.06	< 0.05	< 0.05	< 0.05
3.0 b-wave	29.65 ± 1.99	17.4 ± 1.44	16.89 ± 1.04	10.9 ± 1.61	< 0.05	< 0.05	< 0.05

Considering the effect of intraocular pressure (IOP) on the mouse retina, we checked the IOP at the 6th week and 32nd week in each group with an Icare tonometry (Icare ic200, NC, USA). No significant changes were shown in IOP among all of the groups.

### HFD or *Apoe* deficiency alone induced apoptosis of RGCs in the retina

**Figure 8:**
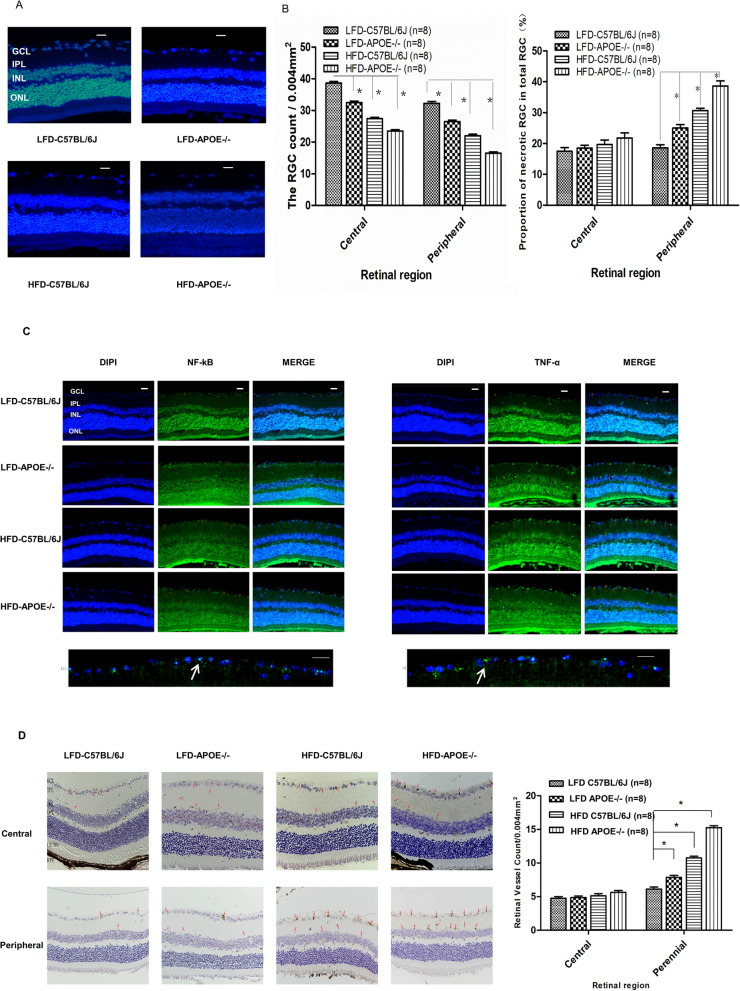
HFD or apoE deficiency alone induced apoptosis of RGCs and vascular angiogenesis in retina. **A** HFD and apoE deletion decreased the density of Hoechst 33258-positive RGCs in the retina. Rats retinas from different groups were harvested at 32nd and subjected to whole mount immunostaining. Representative images from control, LFD*-APOE−/*−, HFD-C57BL/6J, HFD-*APOE−/−* group were stained by Hoechst 33258 (blue). Scale bar = 100 μm. **B** Densitometric analysis of the survival of the RGCs (Left) and proportion of necrotic RGCs (Right) in the GCL, which was assessed by counting the number of fluorescent Hoechst 33258 stained RGCs. Data were shown as mean ± SEM (n = 8, per group, *P < 0.01). **C** HFD and apoE deletion induced the activity of NF-κB and TNF-α in the peripheral areas in the retinal ganglion cells layer (GCL). Mice retinas from control, LFD-*APOE−/−*, HFD-C57BL/6J, HFD-*APOE−/− *group were stained by anti-NF-κB (green), anti-TNF (green) and DAPI (blue) Scale bar = 100 μm; Representative images from HFD-*APOE−/− *group (below), Scale bar = 50 μm. **D** HFD and apoE deletion increased the expression of VERGR2 in the peripheral areas in the retinal ganglion cells layer (GCL). The expression of VERGR2 was analyzed by H&E staining. Data of bar graph showed that compared with the VERGR2 expression in central area, a significant changes in the peripheral areas, especially in HFD-*ApoE−/− *group. The results were mean ± SEM. ***P < 0.001 (n = 8). Scale bar: 500 μm. *GCL* ganglion cell layer, *IPL* inner plexiform layer, *INL* inner nuclear layer, *OPL* outer plexiform layer, *ONL* outer nuclear layer, *CHO* choroid.

To further identify the reasons for these changes, we first stained RGCs with Hoechst 33258 and regarded the neurons with fragmented or reduced nuclei as apoptotic cells. The survival of the RGCs was evaluated on retinal flat mounting at the 32nd week in the different groups. As shown in Fig. 8A, the number of Hoechst 33258-stained RGCs decreased in groups of LFD *Apoe*^−/−^ mice, HFD C57BL/6J mice and HFD *Apoe*^−/−^ mice in an area of 0.004 mm^2^ compared with the LFD C57BL/6J mice (LFD C57BL/6J: 43.13 ± 2.03/0.004 mm^2^, n = 8; LFD *Apoe−/−*: 29.75 ± 2.38/0.004 mm^2^, n = 8; HFD C57BL/6J: 24.88 ± 1.36/0.004 mm^2^, n = 8; HFD *Apoe−/−*: 17.00 ± 1.41/0.004 mm^2^, n = 8). The data showed that, compared with the control group (18.55%), the apoptotic RGCs of the LFD *Apoe−/−* and HFD C57BL/6J mice accounted for 33.61% and 38.69%, respectively. More Hoechst 33258-stained RGCs were observed in the HFD *Apoe−/−* mice (49.26%) compared with the normal group (all n = 8, *P* < 0.001). To further verify whether apoptosis of RGCs in the peripheral retina was more significant than in the central retina, the differences in the proportions of necrotic RGCs among total RGCs between these two regions were determined. This analysis revealed that there was no significant difference in the proportion of necrotic RGCs in the central retina among the four groups (LFD C57BL/6J: 17.48 ± 3.38%; LFD *Apoe−/−*: 18.46 ± 2.73%; HFD C57BL/6J: 19.63 ± 4.24%; HFD *Apoe−/−*: 21.81 ± 4.62%, one-way ANOVA, F = 1.868 *P* = 0.158 > 0.05). In contrast, analysis of the peripheral retina showed a dramatic increase in the proportion of necrotic RGCs in total RGCs, with all of them being significantly higher in the HFD *Apoe*^−/−^ mice, HFD C57BL/6J mice and LFD *Apoe*^−/−^ mice than in the control group (LFD C57BL/6J: 18.60 ± 2.84%; LFD *Apoe−/−*: 24.98 ± 3.13%; HFD C57BL/6J: 30.68 ± 2.13%; HFD *Apoe−/−*: 38.64 ± 4.65%, all n = 8, Area(central:peripheral) two-way ANOVA F [1, 32] = 60.098 *P* = 0.000 < 0.05; groups one-way ANOVA F = 54.484 *P* = 0.000 < 0.05 as shown in Fig. 8B).

NF-κB is an important nuclear transcription factor involved in inflammatory responses and the regulation of apoptosis. Increased NF-κB activates more inflammatory cytokines, promoting cell apoptosis. Compared to in the normal control group (NF-κB-positive cells (%): LFD C57BL/6J: 11.85 ± 4.17, n = 8), the numbers of NF-κB-positive cells in the RGCs in the HFD C57BL/6J mice (26.37 ± 4.05, n = 8) and LFD *Apoe*^−/−^ mice (24.56 ± 43.96, n = 8) were significantly increased (*P*1 < 0.0001; *P*2 < 0.0001). Under the two factors together (HFD *Apoe*^−/−^ mice), more NF-κB-positive cells in the RGCs were observed (40.36 ± 5.89, n = 8) (*P*4 < 0.0001). The increased NF-κB in the RGCs activated the expression of downstream TNF-α. The TNF-α expression in the retinal NFL + GCL + IPL composite layer of the HFD C57BL/6J mice and the LFD *Apoe*^−/−^ mice was increased, especially in the HFD *Apoe*^−/−^ mice (Fig. 8C). Further, RT-PCR analysis confirmed that the high-fat diet and *Apoe* gene knockout induced TNF-α mRNA levels that were significantly increased compared to those in the normal control group (Fig. S1). The increase in inflammatory agents directly acted on RGCs to aggravate cell death.

In addition, as we mentioned, neovascularization in the retinal vasculature was noted among the different groups. It is known that retinal neovascularization is directly related to vascular endothelial growth factor (VEGF) expression, The latter caused angiogenesis and increased vascular permeability. We further examined the expression of VEGFR2 in the central and peripheral areas of the retina. With hematoxylin–eosin (H&E) staining, there were no differences in the expression of VEGFR2 in the HFD *Apoe*^−/−^ mice, LFD *Apoe*^−/−^ and HFD C57BL/6J mice compared to the normal control group in the central retina. Interestingly, in the peripheral area of the retina, the expression levels of VEGFR2 in the LFD *Apoe*^−/−^ and HFD C57BL/6J mice (LFD *Apoe*^−/−^: 8.00 ± 0.76/0.004 mm^2^, n = 8; HFD C57BL/6J: 11.13 ± 1.13/0.004 mm^2^, n = 8) were significantly increased compared to those in the LFD C57BL/6 mice (6.00 ± 0.93/0.004 mm^2^, n = 8) (*P*1 = 0.001, *P*2 < 0.0001). In the HFD *Apoe*^−/−^ mice, the increase in VEGFR2 expression was more significant (*P*4 < 0.0001) (Fig. 8D).

The results showed that both HFD and *Apoe*^−/−^ increased the expression of NF-κB in the RGCs and activated the expression of downstream TNF-α, further promoting RGC apoptosis. No further changes in VEGFR2 expression in the central retina were induced by either a single factor or two factors. However, HFD or *Apoe*^−/−^ increased the expression of VEGFR2 compared to the normal controls, and the expression of VEGFR2 increased more notably under the two factors together in the peripheral retina.

## Discussion

As we known, HFD animals have been used as models to study type 2 diabeticmellitus (T2DM), metabolic syndrome, and obesity for many years^2,11,12^. In recent years, the effect of HFD on the retinal structure and function has become a popular topic^13^. In the human body, the *APOE* gene is located on chromosome 19^14^. APOE is a lipid-related protein that is present in both the serum and CNS^15^. As a ligand for low-density lipoprotein receptors, it is involved in chylomicron and very low-density lipoprotein clearance, and it maintains the body lipid metabolism balance. This protein not only plays an important role in lipid metabolism but also participates in other important biological functions, such as immune regulation and neurological pathway regulation (neuron repair and remodeling). Therefore, in the population, *APOE* gene abnormalities could lead to cardiovascular^16^, cerebrovascular and nervous system-related diseases^17^. For these reasons, this gene has become a new target for basic research and clinical treatment^18^. *Apoe*-deficient (*Apoe*^−/−^) mice spontaneously developed hypercholesterolemia, atherosclerosis and retinopathy^6^. The effect of cholesterol abnormalities caused by *Apoe* gene deletion on the retina has also been a research focus.

In our study, we used the HFD-fed mice and the *Apoe* gene knockout mice to observe the effects of diet and genes on the structure and function of the retina. First of all, compared with wild-type mice fed a normal diet, the body weight and the amount of VAT in the HFD C57BL/6J and HFD *Apoe*^−/−^ mice were significantly increased. In the HFD *Apoe−/−* mice, serum lipid and systemic glucose levels were highest among the four groups. Although the cholesterol levels of the HFD C57BL/6 and LFD *Apoe*^−/−^ mice were higher than in the control group, they did not achieve the level of the HFD *Apoe*^−/−^ mice, consistent with previous studies by Plump and Zhang^19,20^. Interestingly, due to gene defects, the blood lipids and blood glucose of the LFD *Apoe*^−/−^ mice were higher than in the control group, while the body weights were similar to those in the control group. These results indicated that hyperglycemia or hyperlipemia did not necessarily go hand in hand with obesity. Then, we measured the retinal lipid profile. We found that the retinal sterol profile was elevated, especially in HFD-*ApoE−/− *mice. As a negative regulator, no significant up-regulation of SCAP-SREBP expression was observed. These results were consistent with Nicole El-Darzi’s study^21^.

Next, we further demonstrated that a 32-week feeding period of the high fat diet to *Apoe*-deficient mice and wild-type C57BL/6 mice resulted in dramatic reduction in the a- and b-wave amplitude of ERG compared to that in regular mice, indicating that HFD impacted the function of the retina. These results were consistent with the results of most studies^22,23^. Some other studies showed that only decreased amplitude of the a- and b-waves prolonged the latency of ERG as seen in the HFD *Apoe*^−/−^ mice, whereas the retinal light response of the HFD C57BL/6J and LFD *Apoe*^−/−^ mice was similar to that of the normal control group (LFD C57BL/6J)^22^. Controversially, in the present study, there was still apparently decreased a- and b- wave's amplitude of ERG in the LFD *Apoe*^−/−^ mice compared with that of the normal control group, although the reduced amplitude of ERG was not as significant as in the HFD *Apoe*^−/−^ mice. This outcome suggested that hypercholesterolemia might play a major role in damage to neuroretinal cells and impairment of retinal function among these risk factors.

Third, to distinguish different thicknesses between different regions, we examined each layer thickness of the central and peripheral retina using SD-OCT^24^. We found that, for the central retina, diet and gene deficiency have little effect on the thickness of each retinal layer. This result agreed with the study by Ritland JS^25^, perhaps because of the rich blood supply, good nutrients and lack of aggregation of inflammatory cytokines in the central area. This hypothesis was confirmed in our subsequent findings on fundus fluorescence angiography (FFA), which showed fewer changes occurring in blood vessels (vascular area, percentage of vascular retina, number of total branch points and nonperfused area) in the HFD or *Apoe*^−/−^ group than in the normal control group in the central retina. In the peripheral retina, the thickness of the NFL + GCL + IPL composite layer in the HFD and *Apoe*^−/−^ mice was significantly thinner, and that in the HFD *Apoe*^−/−^ mice was much thinner. Since the retinal vessels are mainly located in the NFL layer, FFA tests were more sensitive in detecting the vessel distribution and blood perfusion in this specific structure. These OCT changes were also supported by the FFA results. We found that the percentage of vascular retina and number of total branch points in the HFD C57BL/6J, LFD *Apoe*^−/−^ and HFD *Apoe*^−/−^ mice were all increased. These findings might be related to the microvessels supplying the peripheral retina, rendering the peripheral retina more prone to ischemia and hypoxia. It could not excrete metabolites in a timely manner and received less nutrition in this state. Because of rod cells mainly located in the peripheral retina, night blindness can also occur when photoreceptors dysfunction.

The recent study by Bright Asare-Bediako revealed that the HFD mouse is a useful model for examining the effects of prediabetes and hypercholesterolemia on the retina^26^. Interestingly, the severity of retinopathy depended on HFD feeding duration^12^. It was considered that retinal neovascularization occurs in mice after 7 months HFD feeding^27^. Bright Asare-Bediako et al.^26^ found that C57BL/6 mice fed with HFD showed a reduction in a-wave and b-wave amplitudes at 6 months and increased retinal nerve infarcts and vascular leakage, as well as reduced vascular density at 12 months. This evidence supported our findings. The HFD-induced changes appeared to occur slower than those observed in T2DM models but were consistent with other retinopathy models, showing neural damage prior to vascular changes. This result falled in line with our findings^26^. We confirmed that a 32-week HFD (8 months) could induce both retinal vascularization and neurologic changes.

Finally, the underlying mechanism for vascularization and neurologic changes were detected. A large aggregation of studies has focused on underlying mechanisms caused by HFD. Mykkänen et al. believed that 12-week HFD feeding could induce differential products of a variety of stress-related genes in the retina, such as nitric oxide synthase and hydroxynonenal, leading to retinal degeneration^28^. The other study suggested that changes in the diabetic retinal fundus caused by HFD were due to decreased glucose tolerance and enhanced insulin resistance^29^. These pathological changes were related to TLR4-dependent macrophages or microglia activation and perivascular oxidative stress marker upregulation^11^. Chang et al. observed that 3–6 months of HFD feeding impaired retinal function through calcium signaling abnormalities and microglial activation. The reduction in ERG components in early diabetic retinopathy reflected the reduction in neuronal activity in retinal light responses, which might have occurred due to decreased calcium signaling transduction in neurons based on changes in PI3K-Akt signaling in L-type voltage-gated calcium channels and plasma membrane Ca^2+^-ATPase pathway regulation ability^30^.

APOE contains four subtypes, and type 4 (Apoe4) works on blood vessels, which might be associated with VEGF expression downregulation^31^. Hypercholesterolemia caused by *Apoe* gene knockout partially or completely blocked the retinal vessels and upregulated the expression of VEGF, which resulted in dramatic retinal neovascularization^23^. All of the aforementioned findings indicated that APOE has a certain protective effect on retinal vessels. Chrysostomou et al. also suggested that the outward growth of glia-related synapses seems to be mediated by *Apoe* partially^32^. *Apoe* deletion led to a decrease in the number of activated glial cells and a reduction in the outward growth of RGC nerve processes, hinting that *Apoe* had a protective effect on retinal nerve cells^33,34^. Drouet et al.^35^ pointed out that lipid-bound *Apoe*, not lipid-free *Apoe*, can protect neurons from apoptosis. They believed that *Apoe* was necessary for the prevention of RGC apoptosis. This protective effect of *Apoe* called for lipids, but no cholesterol was required. Their research showed that the cholesterol-fed *Apoe* gene knockout mice had retinal dysfunction (decreased ERG), while little or no calretinin immunoreactivity (neuron-specific calcium binding protein) was found in the layers of INL and GCL in the cholesterol-fed *Apoe*^−/−^ mice, which indicated that the neural retinal cells of the cholesterol-fed *Apoe*^−/−^ mice were undergoing cell death. It was believed to be related to the upregulation of the Bax immune response^22^. In addition, compared to the HFD *Apoe*^−/−^ mice fed cholesterol for 25 weeks, the retinal abnormalities in the cholesterol-fed *Apoe*^−/−^ mice for 35 weeks were more obvious, suggesting that the retinal degeneration in the *Apoe*^−/−^ mice is supposed to be associated with the cholesterol feeding duration^22^. Furthermore, different macronutrient compositions of high-fat diet should be considered as key factors which may lead to the retina impairment in varying degrees. Therefore, it was reasonable to assume that the retinal dysfunction was due to the combination of several pathological conditions (e.g., hypercholesterolemia, atherosclerosis, hypertension and *Apoe* deficiency). Conversely, the opposite view also exists. Gregory et al. suggested that the increased loss of ganglion cells in the retina was not affected by the *Apoe* gene^25^.

It was believed that HFD and Apoe deficiency-induced retinopathy is a type of preproliferative diabetic retinopathy, in which the death of perithelial cells and endothelial cells leads to retinal ischemia^36^. However, the reasons for apoptosis in ganglion cells remain poorly understood. We proposed the following question: As a continuation of the CNS, the retina is rich in blood vessels and nerves. Does an HFD result in increased inflammation response, while *Apoe* gene deletion leads to the increased sensitivity of retinal nerve cells to ischemic injury? To further explore the causes of the thinning of the NFL + GCL + IPL composite layer and increased vascular area, nonperfused area and hemangiomatous area in HFD and *Apoe*^−/−^ mice, we conducted the following steps.

First, we detected the number of apoptotic RGCs in the peripheral and central retina. RGCs were stained with Hoechst 33258, and the neurons containing fragmented or reduced nuclei were regarded as apoptotic cells. It was demonstrated that the percentage of apoptotic RGCs in hypercholesterolemic mice induced by HFD and *Apoe*^−/−^ out of the total number of neurons was increased in the peripheral retina compared to the central retina. Second, as we know, NF-κB is an important nuclear transcription factor. It participates in the body's inflammatory reaction and immune response and can regulate apoptosis and the stress response^37^, so we examined NF-κB expression in the NFL + GCL + IPL composite layer and found that it was significantly higher in the HFD and *Apoe*^−/−^ mice than in the normal control group. Puig et al. showed that HFD can increase TNF-α and thus activate microglia and macrophages in the mouse brain^38^. To confirm the role of TNF-α in these conditions, we investigated the expression of the corresponding inflammatory factor, TNF-α, regulated by NF-κB, which was also increased in this particular layer. Then, we confirmed that the increased expression of TNF-α in the retina is directly related to the upregulated expression of TNF-α mRNA. The inflammatory responses initiated through NF-κB and TNF-α resulted in the apoptosis of RGCs. In the other hand, we further detected VEGFR2 levels, which caused angiogenesis and increased vascular permeability. The results showed that the expression of VEGFR2 was higher in the HFD and *Apoe*^−/−^ mice than in the control group, in agreement with Sheng’s findings^8^. He indicated that CNS neurons of the *Apoe*^−/−^ mice were more sensitive to ischemic injury than wild-type neurons under a normal diet^8^. Liu’s study^39^ suggested that HIF-1α plays an essential role in systemic responses to hypoxia and targets VEGF. Therefore, it might be a choice to evaluate HIF-1α expression. It is worth noting that a single factor (such as HFD or *Apoe*^−/−^) caused retinal injury far less than that caused by combined factors (HFD + gene abnormality). In summary, inflammatory response is considered as a contributor to chronic retinopathy. Herein, We prove the hypothesis: these changes appears to be the inflammatory responses initiated through NF-κB and TNF-α, resulted in apoptosis of RGCs. Also, increased VERFR2 levels caused angiogenesis and increased vascular permeability.

Due to the increasing global population and improved living conditions, the number of people with HFD and hypercholesterolemia is growing remarkably, and related systemic diseases have become a major social issue of global concern. This study showed that a high-fat diet could lead to prediabetic retinopathy, related to the duration of HFD feeding. For the population with *APOE* abnormalities, a reasonable dietary structure can delay or alleviate the retinal structural and functional changes. However, because of genetic predispositions, for those people without dietary control, retinal dysfunctions rapidly progress and are difficult to control, which can severely impact quality of life.

## Materials and methods

### Animals

Male mice (C57BL/6J, wild-type and *Apoe*^−/−^) were used in this study. All *Apoe*^−/−^ mice have the same genetic background as C57BL/6J mice. All of the experimental mice were purchased from Beijing Vital River Laboratories Co., Ltd. [SCXK (JING) 2016-0006, Beijing, China] and fed in the laboratory animal room of Tianjin University of Science and Technology (temperature 22 °C, relative humidity 40–60%) with a light:dark cycle of 12:12 h. Starting from the 6th week, a HFD (36.72% fat, 13.19% proteins, 50.09% carbohydrates) and a low-fat diet (LFD, control, 7.16% fat, 15.90% proteins, 81.07% carbohydrates) were given to two groups of *Apoe*^−/−^ mice^40^. Two groups of wild-type mice also received the HFD and LFD (control groups). During the experiment, body weight and food intake were monitored, and body weight was measured weekly. After 32 weeks of feeding, the tail veins of the mice were used to examine blood glucose, mouse epididymis tissues were collected, and the serum lipids of the mice were measured to evaluate obesity (Fig. 1A)^41^. All of the animal procedures in this study were approved by the Institutional Animal Care and Use Committee of Nankai University and complied with the ARVO Statement for the Use of Animals in Ophthalmic and Vision Research.

### Electroretinography (ERG)

Retinal function was evaluated with ERG following a previously described procedure. Recording was conducted in a dark room after at least 24 h of adaptation. Thirty-eight-week-old mice were anesthetized by intraperitoneal injection of 5% chloral hydrate (purchased from the General Hospital of Tianjin Medical University, 0.01 mL/g body weight) and were placed on a heating pad to keep the body temperature at 37 °C. Proparacaine hydrochloride eye drops were applied for ocular surface anesthesia. The pupils were dilated with tropicamide 0.5% half an hour before recording. A drop of 1% carbomer gel was used to keep the eye moist to ensure electrical contact and to protect the cornea. Subcutaneous needle electrodes were placed at the back and the tail as reference and ground electrodes, respectively. A loop wire, as an active electrode, was gently positioned on the center of the cornea to record signals. All of the procedures were performed under dim red light. Full-field ERGs were recorded with a visual electrophysiology system (RETI-port/scan 21, Roland Consult Co., Ltd., Brandenburg, Germany). A series of stimulus intensities (0.01, 3.0 and 10.0 cd s/m^2^) was applied for dark-adapted ERGs. Light-adapted ERGs were recorded to stimuli of 3.0–10.0 cd s/m^2^) superimposed on the background light after light adaptation. The amplitude of the a-wave and b-wave was measured from the baseline to the first trough and from the first trough to the next peak and were analyzed using ERG View software (Roland Consult Co., Ltd., Brandenburg, Germany).

### Fundus photography and fundus fluorescein angiography (FFA)

Digital color fundus photographs were obtained using a MICRON IV comprehensive system for rodent retinal imaging (Phoenix Research Labs, Pleasanton, CA, USA) after pupil dilation. Anesthesia and pupil dilation were induced as described above. For FFA, the mice were injected intraperitoneally with 10% sodium florescence dye at a dose of 0.01 mL per 5–6 g body weight, and fundus images were obtained using MICRON IV. FFA images include the central and peripheral regions. The central region is defined as three times the diameter of the optic disc, and the rest of the retina is considered the peripheral region (Fig. 3A). Retinal vessel density was calculated using Angio Tool software (National Institutes of Health National Cancer Institute, Gaithersburg, MD), including the vascular area, percentage of the vascular retinal area, total number of branch points and retinal filling area (as opposed to the nonperfused area)^10^.

### Optical coherence tomography (OCT)

Anesthesia and pupil dilation were induced as described above. During image acquisition, the mouse cornea was always kept moist with the application of carbomer gel to avoid corneal opacity. Image-guided spectral-domain OCT images 2 μm in resolution were obtained using InSight software (Phoenix Research Labs, Pleasanton, CA). The upper, upper-middle, middle, middle-lower and lower areas of the retina were scanned as follows. The scanning lines were positioned 3 PD and 1.5 PD from the optic disc separately. When measuring the central area, a full-length retinal scan was performed across the middle of the disc (Fig. 3C a–e). The retina can be divided into eight regions for the measurement of retinal thickness of each layer: the central superior (CS), central nasal (CN), central inferior (CI), central temporal (CT), paracentral superior (PS), paracentral nasal (PN), paracentral inferior (PI) and paracentral temporal (PT) regions^13^. The sizes of the optic disc, central retina and peripheral retina were 0.2 mm, 0.6 mm and 1.2 mm, respectively (Fig. 3C(f))^24^.

### Immunohistochemistry and immunofluorescence staining

Immunohistochemistry was performed as follows. Eyeballs were enucleated and fixed with formalin (with 5% acetic acid) overnight. After placement in 0.1 M PBS, the eyes were subsequently placed in a gradient of ethyl alcohol and xylene for dehydration before embedding in liquid paraffin. Tissue sections 4 μm in thickness were cut using a cryostat (Microtome Cryostat Microm HM525; Thermo Fisher Scientific, Walldorf, Germany). After deparaffinization and rehydration, the sections were blocked with 10% goat serum for 30 min. Then, the sections were incubated with primary antibody overnight at 4 °C. After triple washing with PBS for 5 min, the slides were incubated with secondary antibody for 1 h. Then, a series of hematoxylin staining and mounting was performed.

For immunofluorescence staining, tissue sections on slides were fixed with 10% goat serum and then incubated with a single primary antibody for single immunofluorescence staining for 1 h. The slides were washed three times with 1 × PBS before incubation with DAPI counterstaining for 30 min. To detect apoptosis, RGCs were stained with Hoechst 33258 (Thermo Fisher Scientific, Waltham, MA, USA) and Ultra Cruz (Santa Cruz Biotechnology, Dallas, TX, USA) mounting medium. The slides were washed three times with 1 × PBS before incubation with a respective secondary antibody for single immunofluorescence staining or two different respective secondary antibodies for double immunofluorescence staining: anti-NF-kB p65 antibody (1:100) [E379] (ab32536), anti-TNF alpha antibody (1:500) [52B83] (ab1793) [Abcam Cambridge, MA, USA]; anti-VEGF antibody (ABS82 ) (1:200) [Sigma St. Louis, MO, USA]. All of the sections were imaged with a Leica DM IRE2 microscope (Leica Microsystems, Bannockburn, IL). Cell counting was performed by two independent masked investigators (CXP & GYB) with a computerized image-analysis system (Image Pro Plus Version 6.0; Media Cybernetics, Silver Spring, MD).

### Quantification of retinal sterols

Mice were anesthetized via intraperitoneal injection of 80 mg/kg ketamine and 15 mg/kg xylazine and sacrificed by cervical dislocation. After eyeballs were enucleate, the retina was carefully taken out. Samples of the washed retina of the same genotype were combined and homogenized. Sterols were quantified by isotope dilution gas chromatography-mass spectrometry (GC–MS), as described(Tianjin University of Science and Technology). Unesterified cholesterol and total cholesterol were measured by GC–MS with deuterated sterol analogs as internal standards^42^.

### Reverse transcription, quantitative real-time polymerase chain reaction

Total RNA was isolated from the retina using TRIzol reagent (Invitrogen, CA, USA) according to the manufacturer’s instructions. Quantitative real-time PCR (qPCR) was performed on an Mx3000P (Agilent Technologies) using gene-specific primers for GAPDH, TNF-α, SCAP and SREBP-1 (Table 3) and Power SYBR Green PCR Master Mix (Applied Biosystems). GAPDH was used as an internal loading control to normalize all of the PCR products. The band intensities of the amplified DNAs were compared after visualization on a UV transilluminator.

**Table 3:** RT-PCR probes ID and sequences.

Primer	Sequences	Size (bp)
GAPDH	f-AGGCCGGTGCTGAGTATGTC	530
	r-TGCCTGCTTCACCTTCT	
TNF-α	f-AGTTCTATGGCCCAGACCCT	463
	r-CGGACTCCGCAAAGTCTAAG	
SCAP	f- GGCAATCTC ATCGTGG	369
	r-ATGGTCTTGGCTCCCT	
SREBP-1	f-TGGAGACATCGCAAACAAG,	274
	r-GGTAGACAACAGCCGCATC	

### Data analysis

All of the data are expressed as the mean ± standard error of the mean (SEM). Figures and statistical analysis were performed using GraphPad Prism software (GraphPad Prism 5, GraphPad Prism Software, Inc., San Diego, CA, USA). One-way ANOVA followed by Bonferroni’s correction was used when multiple groups were compared. *P* < 0.05 was considered statistically significant.

## Supplementary information

Supplementary Figure S1.

Supplementary Figure Legend.
