# Supplementary figures and images for: Effects of high-fat diet and Apoe deficiency on retinal structure and function in mice
Source: Sci Rep. 2020 Nov 2;10:18601. doi: 10.1038/s41598-020-75576-7 (PMC7606505; doi:10.1038/s41598-020-75576-7)

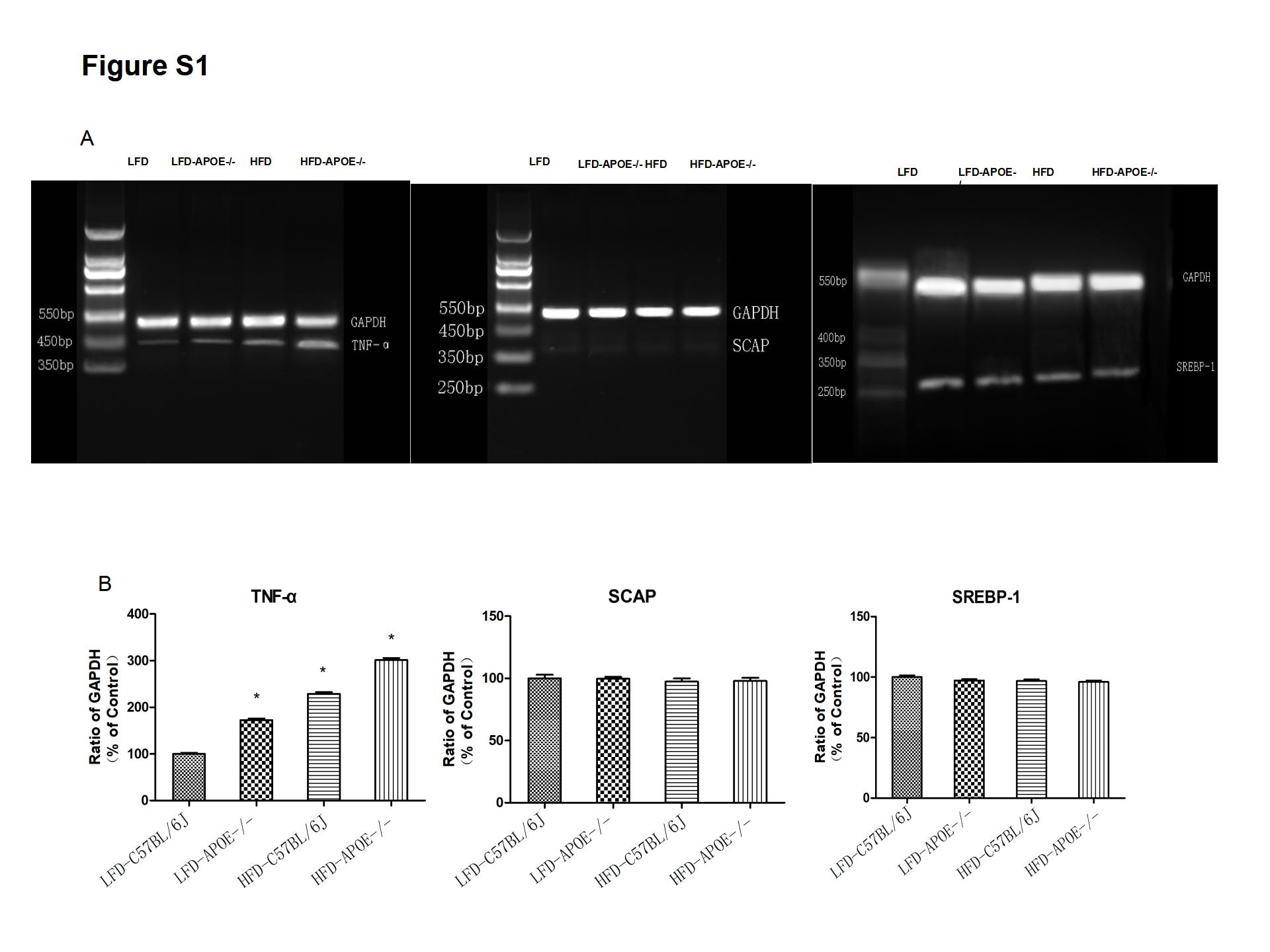

Supplement: Supplementary file 1 — Supplementary Figure S1. [file 41598_2020_75576_MOESM1_ESM.jpg]
