# Supplementary material for: Effects of high-fat diet and Apoe deficiency on retinal structure and function in mice
Source: Sci Rep. 2020 Nov 2;10:18601. doi: 10.1038/s41598-020-75576-7 (PMC7606505; doi:10.1038/s41598-020-75576-7)
Supplement: Supplementary file 2 — Supplementary Figure Legend. [file 41598_2020_75576_MOESM2_ESM.docx]

**Effects of high-fat diet and** ***Apoe*** **deficiency on retinal structure and function in mice**

**Xiupeng Cao^1,2,3#^, Yatu Guo^2,3#^*, Yuchuan Wang^2,3^ , Hao Wang^4^, Dong Liu^4^, Yibo Gong^1,2,3^, Jue Wang^1,2,3^, Xia Chen^1,2,3^*,Wei Zhang^1,2,3^***

**Figure S1 The expressions of lipid metabolism related genes and** TNF-α **in mice retina**

The mRNA expression of tumor necrosis factor (TNF-α)， Sterol cleavage activating protein (SCAP)， sterol regulatory element binding protein (SREBP-1) in Apoe−/− mice and C57BL6/J with HFD and LFD mice. GAPDH was used as the internal reference of PCR. The mean±SEM of 8 cDNA samples were shown in each histogram, and LFD-C57BL/6J was used as the control.*P＜0.05，n=8
